# Performance of point-of-care urine test in diagnosing tuberculosis suspects with and without HIV infection in selected peripheral health settings of Addis Ababa, Ethiopia

**DOI:** 10.1186/s13104-017-2404-4

**Published:** 2017-01-31

**Authors:** Selam Niguse Sahle, Dereje Teshome Asress, Kassu Desta Tullu, Atsebeha Gebrezgeaxier Weldemariam, Habteyes Hailu Tola, Yodit Alemayehu Awas, Gebremdihin Gebremichael Hagos, Muluwork Getahun Worku, Desta Kassa Misgina

**Affiliations:** 1 Medical Microbiology and Immunology Unit, Institute of Biomedical Sciences, Mekelle University, PO. Box: 1871, Mekelle, Ethiopia; 2 College of Health Sciences, Addis Ababa Science and Technology University, Addis Ababa, Ethiopia; 3 School of Medical Laboratory Sciences, Addis Ababa University, Addis Ababa, Ethiopia; 4 HIV/AIDS and TB Research Directorate, Ethiopian Public Health Institute, Addis Ababa, Ethiopia

**Keywords:** Lipoarabinomannan, Point-of-care, Performance

## Abstract

**Background:**

There are few rapid point-of-care tests (POCT) for tuberculosis (TB) for use in resource-constrained settings with high levels of human immunodeficiency virus (HIV). This hinders early tuberculosis (TB) treatment. This cross-sectional study evaluates the recently developed urine Determine tuberculosis lipoarabinomannan (TB LAM) antigen test. A total of 122 participants with signs and symptoms of TB, including 21 (17.1%) participants positive for HIV, were enrolled from September 2011 to March 2012 at three selected health centers in Addis Ababa, Ethiopia. Blood, sputum and urine samples were collected. Löwenstein-Jensen (LJ) solid culture was used as a gold standard to evaluate the performance of the Determine TB LAM antigen test. Data were analyzed using STATA (Statacorp LP, USA).

**Results:**

Of the 122 participants with suspected TB, 35 (28.7%) had TB confirmed bacteriologically by LJ culture. The overall sensitivity, specificity, positive predictive value (PPV) and negative predictive value (NPV) of Determine TB LAM (for both HIV-positive and HIV-negative participants) was 37.1% (95% CI 21.5–55.1), 97.7% (95% CI 91.9–99.7), 86.7% (95% CI 59.5–98.3) and 79.4% (95% CI 70.5–86.6), respectively. However, in participants who were co-infected with TB and HIV, sensitivity, specificity, PPV and NPV were 55.6% (95% CI 21.2–86.3), 100% (95% CI 73.5–100), 100% (95% CI 47.8–100) and 75.0% (95% CI 47.6–92.7). Moreover, the level of immunosuppression of the HIV-infected TB patients was found to have a significant association with the performance of Determine TB LAM (χ^2^ = 7.89, p = 0.002).

**Conclusions:**

The Determine TB LAM test is a potential alternative in peripheral health settings for TB diagnosis in patients who are co-infected with HIV, with advanced immunosuppression.

## Background

The double burden of tuberculosis (TB) and HIV remains the greatest challenge to medicine and public health in developing countries. Ethiopia ranks seventh among the 22 high-burden TB countries [1, 2]. In the absence of an effective vaccine for TB in these countries, the control and ultimate elimination of this disease depends on prompt diagnosis and therapeutic intervention to minimize ongoing transmission [3, 4].

Current tools for TB diagnosis have serious limitations like, poor speed, requiring high skilled experts and infrastructure and poor sensitivity, which cause delays in the diagnosis and treatment of active TB especially in individuals co-infected with HIV infection and smear negatives.

High performing tests are very expensive to apply in resource-limited settings [5–9].

Progress towards TB elimination has slowed. Reversing this decline requires renewed efforts in the scale-up of early diagnosis and proper treatment [10, 11]. This requires prioritizing the development of POCT that are simple, accurate, inexpensive and easily established in resource-limited settings [12, 13]. Alere has recently developed the Determine TB LAM POCT. The test provides results by detecting the LAM antigen in urine samples using the lateral flow principle [14].

Determine TB LAM could useful in providing important augmented sensitivity in settings where smear microscopy remains the only available microbiological test [15]. Though it requires further studies, the performance of the test for diagnosis of TB in HIV-infected individuals is promising [14–19]. This study was conducted to fill the knowledge gap by evaluating the performance of Determine TB LAM for TB diagnosis in Ethiopia.

## Method

### Study setting and study participants

A cross-sectional study was carried out from September 2011 to March 2012 in three selected health centers (Saris, Wereda 6 and Wereda 7) in Addis Ababa, Ethiopia. These healthcare facilities are government-owned organizations that provide public health services including TB and HIV diagnosis and treatment. Consecutively recruited participants were adults (≥18 years) visiting the health facilities with suspected TB who had one of the signs and symptoms of TB (current cough lasting at least 2 weeks, bloody cough, fever, weight loss, chest pain, feeling tired, night sweats, breath shortness), and who were voluntary for HIV testing and were naïve for highly active antiretroviral therapy (HAART) and anti-TB treatment were recruited consecutively. The study excluded participants who were severely ill (with critical conditions and unable to provide consent), those with chronic diseases (such as hypertension, diabetes mellitus, renal failure and cancer, which would act as confounding factors in the performance of the test in HIV-infected patients), pregnant women (pregnancy could be a confounding factor due to immunological shifts during pregnancy) and those under immunosuppressive treatments.

### Sample size

A sample size of 100–200 is recommended for method validation experiments [20]. Therefore following judgment sampling technique, a total of 122 patients with suspected TB were consecutively recruited during the study period.

### Data collection

#### Questionnaires

A structured questionnaire was prepared and socio-demographic data including sex and age, and clinical data on body mass index (BMI) and TB-specific signs and symptoms, were collected by the clinician.

### Laboratory procedures

#### Sample collection

Urine, expectorated sputum and blood were collected from each participant. All samples were transported to the national HIV and TB reference laboratory of the Ethiopian Public Health Institute (EPHI) for laboratory analysis.

#### Laboratory analysis

The Determine TB LAM test was performed by applying 60 μL of fresh urine to the sample pad on the Determine TB LAM Ag strip (Alere international ltd, South Africa) and interpreted following the manufacturer’s instructions. Sputum samples were processed for Löwenstein-Jensen (LJ) culture. Modified Petroff’s method was used to digest and decontaminate the sputum specimens. A 100–200 μL aliquot of the sample was then inoculated into two tubes of LJ medium. The growth of the bacteria was read every week from 3 to 8 weeks according to the Standard operating procedures. Identification of *Mycobacterium tuberculosis* complex from other mycobacterial species was done using TB Ag MPT64 rapid capilia test (Capilia TB-Neo, Japan). Sputum smears were also prepared from the sediment of processed specimen. The slides were coded, dried in air, heat fixed, and stained by the Ziehls Neelson (ZN) technique and examined for acid-fast bacillus (AFB) using 100× oil immersion microscopy. In addition, blood samples were added to tubes containing fluorochrome-labeled antibodies and analyzed using FACS Caliber Flow cytometry analyzer for CD4 determination.

### Statistical analysis

Data were analyzed using STATA version 10 analytical software. Determine TB LAM test sensitivity, specificity, and positive and negative predictive values were defined by comparing results with the gold-standard test for active TB, the sputum LJ culture. The presence of association of independent variables with the Determine TB LAM test performance was evaluated using the Chi squared test. p values less than 0.05 were considered significant.

## Results

### Characteristics of participants

The 122 study participants had a median (IQR) age of 30 (23–45) years; 68 (56%) were male (Table 1). Twenty-one (17.2%) of the participants enrolled were infected with HIV. The mean (standard deviation; SD) CD4 count was 556 (307) cells/µL. The majority (56.6%) of the participants had a body mass index (BMI) in the normal range (18.5 < BMI < 24.99 kg/m^2^). The study participants were primarily screened for signs and symptoms of TB. All the study participants (100%) had a cough lasting at least 2 weeks. Tiredness and chest pain were experienced in 82 and 80% of the study participants, respectively. Weight loss, shortness of breath and coughing with blood were also observed in 67, 63 and 21% of the study participants, respectively.

**Table 1:** Study participant’s demographic and clinical characteristics of TB suspects

Characteristics	Number	Percentage
Sex		
Female	54	44
Male	68	56
Age (year)		
Adults <65	112	92
Adults ≥65	10	8
CD4 count (cells/µL)		
CD4 < 200	24	20
CD4 ≥ 200	98	80
BMI category (kg/m^2^)		
BMI < 18.5	38	30.1
18.5 < BMI < 24.99	69	56.6
BMI ≥25	15	12.3
HIV status		
Positive	21	17.2
Negative	101	82.8
Sign and symptoms for TB		
Current cough ≥2 weeks	122	100
Bloody cough	26	21
Fever	89	73
Weight loss	82	67
Chest pain	98	80
Feeling tired	100	82
Night sweats	85	70
Breath shortness	77	63

**Fig. 1:**
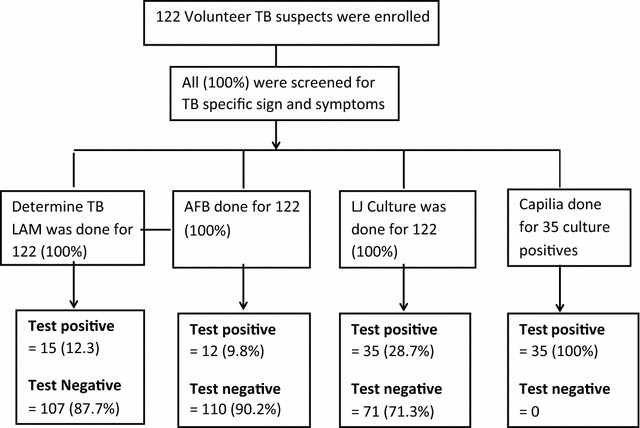
Tuberculosis diagnostic flow and diagnostic results of TB suspects

Of the 122 participants with suspected TB, 21 (17.2%) were HIV positive. Thirty-five participants (28.7%) had TB bacteriologically confirmed by LJ culture. Of the LJ culture positives, 24/35 (68.6%) were sputum smear-negative, and 9 (25.7%) were HIV co-infected (Fig. 1).

### Performance of urine Determine TB LAM test

The strength of the Determine TB LAM test to diagnose TB irrespective of patients’ HIV status was calculated. The Determine TB LAM test was positive in 15 (12.3%) of the 122 study participants and 2 of these 15 were LJ culture negative. We found the sensitivity, specificity, PPV, and NPV of Determine TB LAM to be 37.1% (95% CI 21.5–55.1), 97.7% (95% CI 91.9–99.7), 86.7% (95% CI 59.5–98.3) and 79.4% (95% CI 70.5–86.6), respectively.

We compared the performance of the Determine TB LAM test with the currently available conventional TB diagnostic test at the peripheral level, the AFB smear microscopy. Seven participants who were TB negative by the conventional AFB smear microscopy were positive by the Determine TB LAM test. Of these, 5 were LJ culture positive and 2 were LJ culture negative. Interestingly, when the two tests (AFB smear microscopy and Determine TB LAM test) were combined, 8 individuals were found to be positive and all were LJ culture positive (Table 2). We found significant association between smear status and Determine TB LAM test results (χ^2^ = 36.45, p < 0.001).

**Table 2:** Performance of Determine TB LAM test and smear microscopy, when combined and used alone for the diagnosing TB suspects

	AFB versus culture		Determine TB-LAM versus culture		Determine TB LAM and AFB versus culture	
	Sensitivity% (95% CI)	Specificity% (95% CI)	Sensitivity% (95% CI)	Specificity% (95% CI)	Sensitivity% (95% CI)	Specificity% (95% CI)
All (n = 122)	31.4 (16.9–49.3)	98.9 (93.8–99.9)	37.1 (21.5–55.1)	97.7 (21.5–55.1)	72.7 (39.0–93.9)	100 (2.5–100)

### Performance of the Determine TB LAM test in HIV co-infected participants

**Fig. 2:**
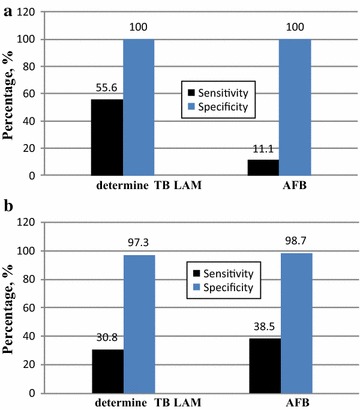
Sensitivity and specificity of Determine TB LAM test and smear microscopy; **a** among HIV co-infected individuals **b** among HIV negative individuals

We also evaluated the effect of HIV infection on the performance of the Determine TB LAM test for TB diagnosis. The sensitivity, specificity, PPV and NPV of the Determine TB LAM test in patients suspected of TB who were co-infected with HIV were 55.6% (95% CI 21.2–86.3), 100% (95% CI 73.5–100), 100% (95% CI 47.8–100) and 75.0% (95% CI 47.6–92.7), respectively (Fig. 2).

Among the 21 patients with suspected TB who were HIV positive, 5 were TB positive by the Determine TB LAM test and 1 was positive by AFB smear microscopy. When the Determine TB LAM test and AFB were compared with the gold standard LJ culture, the Determine TB LAM test was positive in 55.5%, but AFB was positive in 11.1%, of the HIV co-infected TB patients. This indicates the gap in the use of AFB microscopy in HIV-infected TB patients.

Interestingly, we further stratified the 5 HIV-infected individuals who were Determine TB LAM test positive, but AFB smear negative, by their CD4 count (those with <200 cells per µL and those with ≥200 cells per µL)to see if the performance of the Determine TB LAM test was affected by the level of immunosuppression. All 5 HIV-infected individuals with a CD4 count of <200 cells per µL were positive by the Determine TB LAM test, but negative with AFB microscopy. However, the Determine TB LAM test was negative in all of the HIV-infected individuals with a CD4 count ≥200 cells per µL, and AFB was positive in 1 of these. We found significant associations between the performance of the Determine TB LAM test and HIV infection status (χ^2^ = 3.12, p < 0.04) and level of immunosuppression (χ^2^ = 7.89, p < 0.005).

## Discussion

Searching for and evaluating TB diagnostic assays that are easily applied in developing countries challenged by the increased burden of TB and HIV infections is of worldwide importance. The Determine TB LAM test is a lateral-flow rapid point-of-care test developed recently by Alere. This method is developed for use where, due to resource constraints, there is limited availability of advanced assays. Studies evaluating the performance of Determine TB LAM in different geographical locations are limited. We evaluated the performance of this assay in response to this need. As our focus was the performance of the test in HIV-infected patients, we excluded those with factors that could confound the results of the study (chronic disease, pregnancy and immunosuppressive medications).

The overall sensitivity, specificity, PPV and NPV of the Determine TB LAM test to diagnose TB irrespective of patient HIV status found in this study was similar to the findings of recent studies [14–18]. Although AFB smear microscopy remains the conventional diagnostic tool for TB in resource-limited settings, its decreased sensitivity reinforces the need for additional point-of-care assays in these settings. In our study, Determine TB LAM test detected TB in 5 (20.7%) individuals whose TB was missed by the conventional smear microscopy. This strongly suggests that Determine TB LAM may be used to address the challenges in diagnosing TB using AFB microscopy.

In this study, some individuals whose TB was missed by Determine TB LAM were detected as positive by smear microscopy, indicating the value of smear microscopy in diagnosing TB. However, an interesting additive effect occurred when combining the two peripheral diagnostic assays (smear microscopy and Determine TB LAM).

We found higher sensitivity than in a study conducted by Lawn et al. (which was 43.7%). This difference might be due to the fact that they used frozen samples and the effect of freezing urine samples is not well known [14, 15]. In addition to the challenge of diagnosing smear-negative TB, diagnosis of TB in HIV co-infected patients is another challenge to controlling TB in resource-limited settings. Rapid detection of active TB is essential for managing patients with advanced HIV infection as it permits earlier initiation of TB therapy and institution of infection control procedures in high-burden settings. In this study, the sensitivity of the Determine TB LAM test in HIV-infected patients was shown to be much better than in HIV-negative patients. Interestingly, sensitivity was strongly associated with patients’ CD4 cell counts, with the highest sensitivity recorded for those with advanced immunosuppression (CD4 count <200 cells/µL). This was comparable with other findings done on HIV HIV-infected TB patients [14–18].

In relation to this, the sensitivity of the conventional diagnostic assay in high-burden settings (smear microscopy) is much lower compared with Determine TB LAM for diagnosis of TB in HIV-infected patients, especially those with advanced immunosuppression. Moreover studies have also indicated that the performance of Determine TB LAM test was greater in patients with advanced lower CD4 count. This might be related with the fact that HIV-infected patients with advanced immunosuppression have more severe disease and a likely higher antigen burden of lipoarabinomannan antigen in urine [12]. It might also be due to the changes in glomerular filtration secondary to HIV-related podocyte dysfunction [21].

## Conclusion

We found the sensitivity of the Determine TB LAM test increased in patients with HIV co-infection. Thus, the Determine TB LAM test is a promising point-of-care assay for the diagnosis of smear-negative TB patients and HIV-infected individuals, especially those with advanced immunosuppression. Moreover, the combination of smear microscopy and Determine TB LAM tests could improve the diagnosis of TB in peripheral health settings. Further research in different groups including children and in resource-constrained settings in different geographical locations is warranted.

## Abbreviations

AFB: acid fast bacilli
Ag: antigen
BMI: body mass index
HAART: highly active anti-retroviral therapy
HIV: human immunodeficiency virus
LAM: lipoarabinomannan
LJ: lowenstein Jensen
mm: millimeter
NPV: negative predictive value
OPDs: outpatient departments
PPV: positive predictive value
TB: tuberculosis
WHO: World Health Organization
